# Y4 RNA fragments from cardiosphere-derived cells ameliorate diabetic myocardial ischemia‒reperfusion injury by inhibiting protein kinase C β-mediated macrophage polarization

**DOI:** 10.1186/s12933-024-02247-6

**Published:** 2024-06-12

**Authors:** De-Zhao Liu, Xiao-Zhi Luo, Chuang-Hong Lu, Yang-Yi Feng, De-Xin Chen, Zhi-Yu Zeng, Feng Huang

**Affiliations:** Department of Cardiology & Guangxi Key Laboratory Base of Precision Medicine in Cardio-Cerebrovascular Diseases Control and Prevention & Guangxi Clinical Research Center for Cardio-Cerebrovascular Diseases, The First Affiliated Hospital of Guangxi Medical University, No.6 Shuangyong Road, Nanning, 530021 Guangxi China

**Keywords:** Diabetes, Myocardial ischemia/reperfusion injury, Macrophages, Y4 RNA, Protein kinase C β

## Abstract

The specific pathophysiological pathways through which diabetes exacerbates myocardial ischemia/reperfusion (I/R) injury remain unclear; however, dysregulation of immune and inflammatory cells, potentially driven by abnormalities in their number and function due to diabetes, may play a significant role. In the present investigation, we simulated myocardial I/R injury by inducing ischemia through ligation of the left anterior descending coronary artery in mice for 40 min, followed by reperfusion for 24 h. Previous studies have indicated that protein kinase Cβ (PKCβ) is upregulated under hyperglycemic conditions and is implicated in the development of various diabetic complications. The Y4 RNA fragment is identified as the predominant small RNA component present in the extracellular vesicles of cardio sphere-derived cells (CDCs), exhibiting notable anti-inflammatory properties in the contexts of myocardial infarction and cardiac hypertrophy. Our investigation revealed that the administration of Y4 RNA into the ventricular cavity of db/db mice following myocardial I/R injury markedly enhanced cardiac function. Furthermore, Y4 RNA was observed to facilitate M2 macrophage polarization and interleukin-10 secretion through the suppression of PKCβ activation. The mechanism by which Y4 RNA affects PKCβ by regulating macrophage activation within the inflammatory environment involves the inhibition of ERK1/2 phosphorylation In our study, the role of PKCβ in regulating macrophage polarization during myocardial I/R injury was investigated through the use of PKCβ knockout mice. Our findings indicate that PKCβ plays a crucial role in modulating the inflammatory response associated with macrophage activation in db/db mice experiencing myocardial I/R, with a notable exacerbation of this response observed upon significant upregulation of PKCβ expression. In vitro studies further elucidated the protective mechanism by which Y4 RNA modulates the PKCβ/ERK1/2 signaling pathway to induce M2 macrophage activation. Overall, our findings suggest that Y4 RNA plays an anti-inflammatory role in diabetic I/R injury, suggesting a novel therapeutic approach for managing myocardial I/R injury in diabetic individuals.

**Supplementary Information:**

The online version contains supplementary material available at 10.1186/s12933-024-02247-6.

## Introduction

Diabetes mellitus (DM) is a prominent chronic metabolic disorder with significant implications for perioperative complications and cardiovascular health [1]. Research indicates that diabetic individuals are at a heightened risk for myocardial ischemia, with incidence rates 2.45 to 2.99 times greater than those of nondiabetic individuals. Furthermore, diabetic patients exhibit increased vulnerability to myocardial ischemia, with compromised myocardial tolerance to ischemic and hypoxic conditions compared to individuals without diabetes [2, 3]. This phenomenon may be attributed to the persistent low-level inflammation of the cardiac tissue resulting from diabetes [4]. Numerous studies have indicated that elevated blood sugar levels prompt immune cells, notably macrophages, to initiate proinflammatory reactions. Diabetic individuals and monocyte macrophage lines cultured in high glucose environments have shown heightened production of interleukins, monocyte chemokines, and other key cytokines [5].

Cardiosphere-derived cells (CDCs) are a population of cells that persist during the embryonic development of the second heart field [6, 7]. As the heart undergoes maturation, these cells reside within the interstitium of the adult heart and play a critical role in cardiac self-renewal and repair following myocardial injury [8]. RNA sequencing analysis revealed that Y RNA is the predominant RNA species found in extracellular vesicles derived from CDCs [9], with further studies indicating that Y4 RNA is the most abundant RNA species in these vesicles [10, 11]. Y RNAs, a type of noncoding RNA with a length of approximately 100 nucleotides, are implicated in various crucial cellular processes, such as the initiation of DNA replication, inhibition of the RNA-binding protein Ro60 [12], histone precursor mRNA processing, and cellular damage repair [13].

Recent research has indicated that treatment of refractory heart failure patients with CDCs can effectively reverse the upregulation of protein kinase Cβ (PKCβ) [14]. However, the impact of Y4 RNA, which is predominantly found in the outer vesicles of CDCs, on PKCβ expression remains uncertain. In this study, we investigated the potential influence of PKCβ deficiency on macrophage polarization following myocardial ischemia/reperfusion (I/R) injury. By elucidating the role of PKCβ in I/R injury, we observed that Y4 RNA intervention significantly mitigated cardiac damage in diabetic I/R models. The protective effect observed in this study may be attributed to the inhibition of the PKCβ/ERK1/2 signaling pathway by Y4 RNA, leading to alterations in macrophage polarization within the diabetic heart following I/R injury. These results offer novel perspectives and strategies for the management and mitigation of diabetic I/R.

## Methods

### Animals

The PKCβ knockout mice utilized in this research were obtained from Cyagen Biosciences (Jiangsu, China). PKCβ-/- mice were generated through the targeted disruption of exon 3, one of the 17 exons of the transcribed PKCβ gene located on mouse chromosome 7, utilizing CRISPR/Cas9 gene editing technology. PKCβ-/- mice were generated by combining the Cas9 protein and four gRNAs targeting exon 3 flanking introns (gRNA-A1: GGA AGT GGA GCG TCC CCA GCT GG and gRNA-A2: CTA GAT AAC TTT CCT TAG ATA GG; gRNA-B1: GCA GGT GAG GAT GTT TCA TCT GG and gRNA-B2: GAT GAT TAT TAT TAT CTG G and gRNA-A1: GAT GAT TAT TAT TAT TAT TAT TAT TAT TAT TAT TAT TAT TAT TAT TAG G). (GAT GAT TAT TGC AAG CAG TCA GG) were co-injected into fertilized mouse eggs. The injected embryos were transferred into female recipient mice to obtain F0 mice. F0 mice were genotyped and tested using F1/R1 (F1: 5’-AAT GTA AGG CCG TTC AAT GAA AG-3’, R1: 5’-AAT ACT GAG CCA AGA AGT GGA GAA G-3’) primers. Genotyped and sequenced F0 mice were mated with wild-type (WT) mice to obtain genetically stable F1 heterozygous mice. Genotypically correct F1 heterozygotes were mated with each other to obtain PKCβ-/- mice.

The db/db mice, aged 6–8 weeks, utilized in this study were procured from Junke Biological Co. (Jiangsu, China) and were characterized as DM mice. Prior to the commencement of the experiment, all mice underwent a one-week acclimatization period and were provided with standard laboratory housing conditions at 25 ± 2 °C with a relative humidity of 50 ± 15% and a normal photoperiod of 12 h of darkness and 12 h of light. The experimental procedures were ethically approved by the Ethics Committee of Guangxi Medical University and adhered to the ARRIVE guidelines for the ethical use of experimental animals.

### RNA synthesis

Y4 RNA (native and modified forms) was custom synthesized by IBSBIO (Shanghai, China). As reported previously [15, 19], the 56-nucleotide sequence for Y4 RNA is as follows: 5’-GGC UGG UCC GAU GGU AGU GGG UUA UCA GAA CUU AUU AAC AUU AGU GUC ACU AAA GU-3’.

### Mouse myocardial I/R injury model

To induce myocardial I/R injury, the mice were subjected to 40 min of myocardial ischemia followed by 24 h of reperfusion. The mice were anesthetized via intraperitoneal injection of tribromoethanol (0.02 mL/g) and positioned in the supine position for intubation and connection to a small ventilator (model 845, Harvard Apparatus, Germany). The edges of the pectoralis major muscle were obliquely cut, and the pectoralis minor muscle was bluntly separated to expose the heart through the left fourth intercostal space. Subsequent to the ligation of the left anterior descending artery (LAD) using a No. 7 − 0 silk suture, a noticeable alteration in color from red to white was observed in the left anterior myocardium, accompanied by a significant reduction or cessation of ventricular wall motion. After 40 min, the ligature was released, and coronary blood flow was restored. Y4 RNA was injected into the myocardium of db/db mice. Specifically, the aorta was clamped using an aortic cross-clamp, and 100 µl of Y4 RNA was injected into the left ventricular cavity for 20 s. Y4 RNA was incubated with Dharmafect transfection reagent (Dharmacon, USA) in IMDM basal medium (Thermo Scientific, USA) at a concentration of 0.15 µg/g for 10 min at room temperature and then resuspended in 100 µl of IMDM for injection. In contrast, the sham-operated db/db mice and the I/R control mice were injected with equal amounts of Y4 RNA-free transfection reagent (Vehicle) in the left ventricular cavity. Twenty-four hours after reperfusion, all mice were evaluated for cardiac function and then euthanized, and their hearts were harvested for further analysis.

### HE staining and immunofluorescence staining

The excised hearts were fixed in 4% paraformaldehyde for 24 h, subsequently embedded in paraffin, and sectioned into 5 μm slices. These sections were dehydrated using an ethanol gradient and cleared with xylene prior to staining with hematoxylin-eosin (HE). The myocardial samples were subjected to immunostaining with specific antibodies against F4/80 (1:200, Cell Signaling Technology, USA), CD86 (1:200, Cell Signaling Technology, USA), and CD206 (1:100, Abcam, UK) on paraffin sections. DAPI was used to stain the nucleus. The quantification of macrophages was performed through image acquisition using Zeiss confocal microscopy and subsequent analysis with ImageJ software.

### Treadmill

Following a 24-hour reperfusion period, the mice from each experimental group underwent exercise treadmill testing. The initial stage consisted of a speed range of 0–10 m/min for 1200 s, followed by a second stage with a speed range of 10–25 m/min for 1800 s. Subsequently, locomotor ability was assessed after acclimatization training, with continuous electrical stimulation of the running table throughout the acclimatization period. Human-induced repulsive stimulation was administered as needed during the testing process. When the mice reached a state of immobility after remaining in the rear third of the running platform for more than six instances, they were classified as exhausted. The distance covered by each mouse at the point of exhaustion was documented.

### Echocardiographic assessment of cardiac function

Mice were anesthetized using tribromoethanol (0.02 mL/g) by intraperitoneal injection, and cardiac function was assessed by an ultrasound machine Vevo2100 (Visualsonics, Toronto, Ontario, Canada) as described previously. The ejection fraction (EF) was determined automatically by the machine, and fractional shortening (FS) was calculated according to the following equation: ((LVIDd-LVIDs)/LVIDd) × 100.

### Infarct area assessment

The infarct area was determined using Evans blue and 2,3,5-triphenyltetrazolium chloride (TTC) staining. Following a 24-hour reperfusion period, the mice were ventilated with a ventilator, and the left anterior descending artery (LAD) was religated. A 2% solution of Evans blue (Sigma Aldrich, USA) was slowly administered through the right ventricle, followed by clamping of the ascending aorta. Subsequently, the hearts were swiftly excised, stained blue, and frozen at -20 °C for 20 min. The hearts were then sectioned into 4–5 segments located 2 mm below the ligature. Each segment, approximately 1–2 mm in thickness, was dissected along the longitudinal axis of the left ventricle. Normal myocardial tissue appears blue, while ischemic regions exhibit a pink hue. A 1% solution of 2,3,5-triphenyltetrazolium chloride from Sigma‒Aldrich, USA, was applied at 37 °C for 10 min. The extent of infarction is quantified as a percentage of the area at risk (AAR).

### Cell culture

Bone marrow-derived macrophages (BMDMs) and neonatal murine ventricular myocytes (NMVMs) were isolated and cultured as previously reported [17, 18]. Y4 RNA (50 nM) was introduced into BMDMs via transfection with Dharmafect 4 reagent. Following centrifugation and cell counting at a 1:8 ratio using a cell counter, the transfected BMDMs were cocultured in Transwell cell culture dishes with NVNMs. The BMDMs were positioned in the upper layer of the Transwell cell culture dishes, while the NVNMs were located in the lower layer. Following a 24-hour coculture period, the Transwell cell culture dishes were transferred to an anoxic chamber and exposed to high-purity N2 gas in low-sugar, serum-free IMMM for 30 min to induce hypoxia and simulate an ischemic environment for an additional 24 h. Subsequently, the cells were incubated in oxygen-saturated, high-sucrose DMEM supplemented with 10% fetal bovine serum to mimic reperfusion for 48 h before harvesting both the cells and their supernatants.

High-glucose BMDMs were infected with an adenovirus overexpressing PKCβ obtained from Hanbio (Shanghai, China), followed by transfection of Y4 RNA into the PKCβ-overexpressing BMDMs. NMVMs were exposed to UV light at a wavelength of 520 nm for 7 min and then incubated at 37 °C in a 5% CO2 environment. Necrotic cell suspensions were subsequently added to macrophage culture flasks transfected with Y4 RNA after a 2-hour interval. After an overnight incubation period of 24 h, the cells were harvested for RNA and protein extraction as well as flow cytometry analysis.

### Western blot analysis

Total protein was extracted from the hearts of db/db mice and BMDMs through a series of procedures, including determination of protein concentration using a bicinchoninic acid assay kit (Solarbio, China). Subsequently, 30 µg of total protein was separated via SDS‒PAGE and transferred to a 0.45 μm polyvinylidene fluoride (PVDF) membrane. Immunoblotting was performed by stimulating the membrane with specific antibodies against PKCβ (1:1000, Cell Signaling Technology, USA), p-Ser661-PKCβ(1:1000, Mlbio, China), JNK (1:1000, Abcam, UK), p-JNK (1:1000, Abcam, UK), ERK (1:1000, Abcam, UK), p-ERK (1:1000, Abcam, UK), P38 (1:1000, Abcam, UK), p-P38 (1:1000, Abcam, UK) and β-actin (1:10000, Abcam, UK).

### qRT‒PCR

Total RNA was extracted from cardiac tissues and cells. Subsequently, 1 µg of RNA was reverse transcribed into cDNA using a T100 Thermal Cycler (Bio-Rad; USA), and quantitative RT‒PCR was carried out with a CFX96 Total (Bio-Rad; USA) following the provided protocol. All the results were normalized against β-actin expression.

### ELISA

To evaluate the levels of creatine kinase MB (CK-MB) and inflammatory factors in the serum and cell supernatants, enzyme-linked immunosorbent assay (ELISA) assays were conducted using a kit from Mlbio (Shanghai, China). Following a 24-hour reperfusion period, the mice were intubated, and blood samples were obtained from the right ventricle postchest cavity opening. The blood samples were centrifuged at 3000 × g for 15 min to collect the supernatant. Cell supernatants were obtained according to established protocols. Subsequent ELISA analysis was carried out in accordance with the provided instructions.

### Flow cytometry

Macrophages were dissociated and isolated using prewarmed Accutase at 37 °C to generate single-cell suspensions. The isolated cells were subsequently incubated with a FITC-conjugated anti-F4-80 antibody, an APC-conjugated anti-CD86 antibody, and a PE-conjugated anti-CD206 antibody (BioLegend, USA) on ice for 20–30 min for phenotypic sorting. Following the incubation period, the cell samples were subjected to two consecutive washes. The samples were subsequently examined utilizing an Attune NxT flow cytometry apparatus (Thermo Fisher Scientific, USA). The macrophage phenotypes were classified as either proinflammatory (F4-80^+^/CD86^+^) or anti-inflammatory (F4-80^+^/CD206^+^). Subsequently, calibration and compensation of experiments involving unstained and single fluorescent controls were performed manually or automatically.

### Statistics

All quantitative data are presented as the mean ± standard error of the mean (SEM). The results were analyzed using Prism 9.0 software (GraphPad, CA). Normal distribution and homogeneity of variance were tested using the Shapiro–Wilk test and Bartlett’s test, respectively. For data that passed both normality and equality of variance tests, comparisons between two groups were performed using the unpaired 2-tailed Student’s t test, and multiple comparisons were analyzed using analysis of variance (ANOVA) followed by Tukey’s post hoc test. Otherwise, the nonparametric Kruskal–Wallis test was applied, followed by Dunn’s test. *P* < 0.05 was considered to indicate statistical significance.

## Results

### Elevated PKCβ and increased inflammatory response in db/db mice with reperfusion injury

**Fig. 1:**
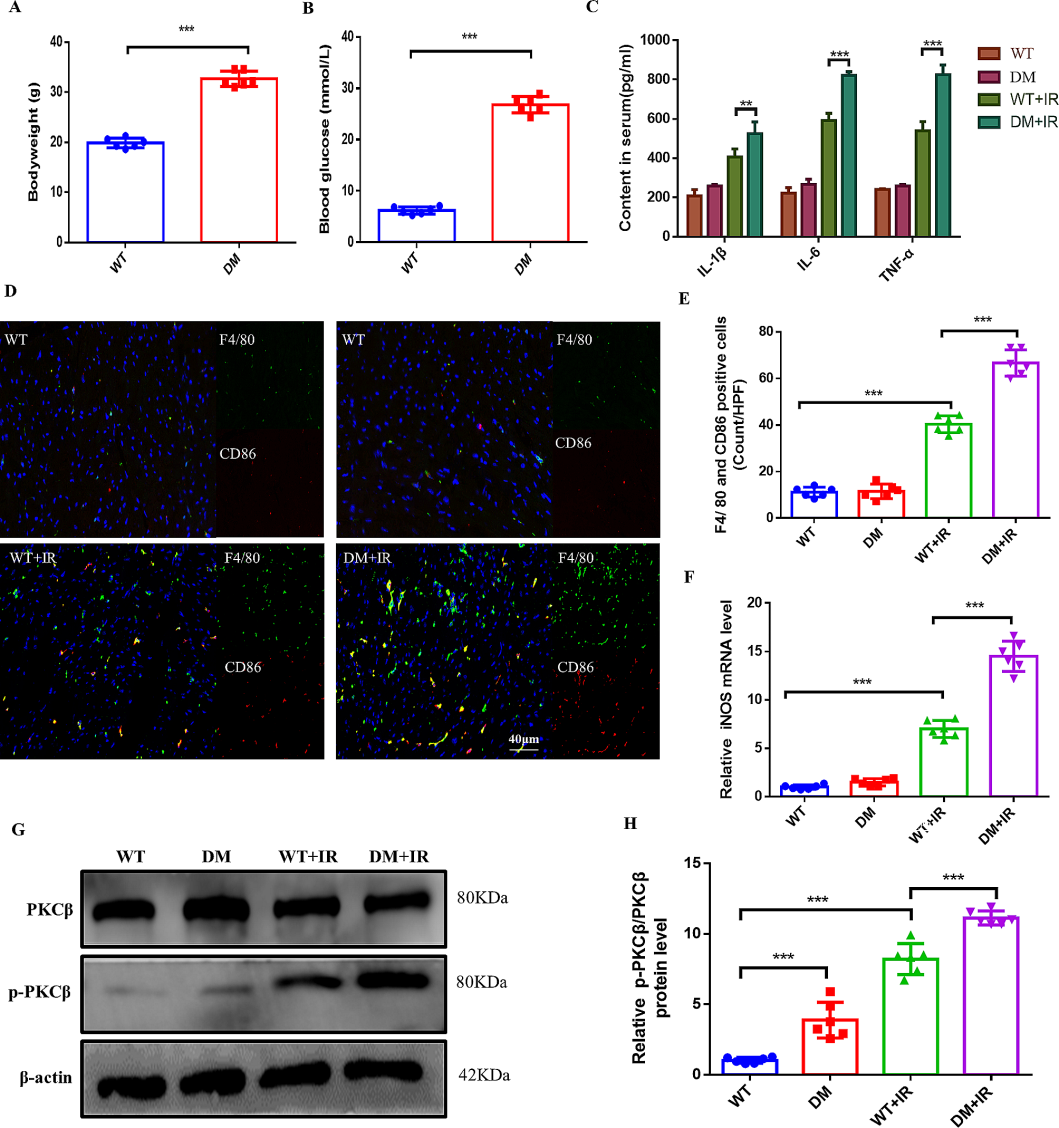
Elevated PKCβ and increased inflammatory response in db/db mice with reperfusion injury.(**A-B**) Body weights and blood glucose levels of WT and DM mice. (**C**)The serum levels of IL-1β, IL-6, and TNF-α in the myocardium of WT and DM mice before and after I/R were determined by ELISA. (**D**) Detection and quantification of M1 macrophages (F4/80+/CD86+) in the hearts of WT and DM mice after I/R by immunofluorescence.(**E**) Quantification of data in D.(**F**) Gene expression of iNOS in myocardial tissue before and after I/R in WT and DM mice was determined by qPCR.(**G**) Representative protein blot of PKCβ and p-PKCβ in the hearts of WT and DM mice before and after I/R.(**H**) Quantification of data in D.*P < 0.05, **P < 0.01, ***P < 0.001; *n* = 6 mice/group

It has been reported that in hyperglycemic mice, worsening of myocardial hypertrophy and fibrosis is positively correlated with enhanced PKCβ expression [19]. Our initial investigation focused on the inflammatory response following myocardial I/R injury in diabetic mice, specifically examining the involvement of PKC β. The db/db mouse model was utilized to represent diabetes, and a mouse model of myocardial I/R injury was established for experimentation. Body weight and blood glucose were significantly greater in db/db mice than in WT mice (Fig. 1A-B). We found that the serum levels of inflammatory factors such as IL-1β, IL-6, and TNF-α were significantly elevated after diabetic cardiac I/R compared with those in the wild type (Fig. 1C). While inflammatory factors were found to be slightly elevated in the serum of db/db mice compared to WT mice in the non-I/R situation, the difference was not deemed statistically significant. We then further examined the distribution of macrophages in the damaged myocardium of mice and found that the number of proinflammatory M1 macrophages was significantly increased (Fig. 1D-E). The confirmation of this conclusion is further supported by the identification of M1 macrophage-specific iNOS using qPCR(Fig. 1F). Subsequently, an analysis was conducted on the expression of PKCβ in the infarcted myocardial tissue of mice within each experimental group. The findings indicated a heightened activation of PKCβ in the myocardial tissues of db/db mice compared to WT mice, even in the absence of I/R injury. Furthermore, following I/R, there was a significant phosphorylation of PKCβ observed in both WT and db/db mice, with a more pronounced activation noted in the db/db mice. (Fig. 1G-H).

### Y4 RNA improves cardiac function after myocardial I/R injury in db/db mice

**Fig. 2:**
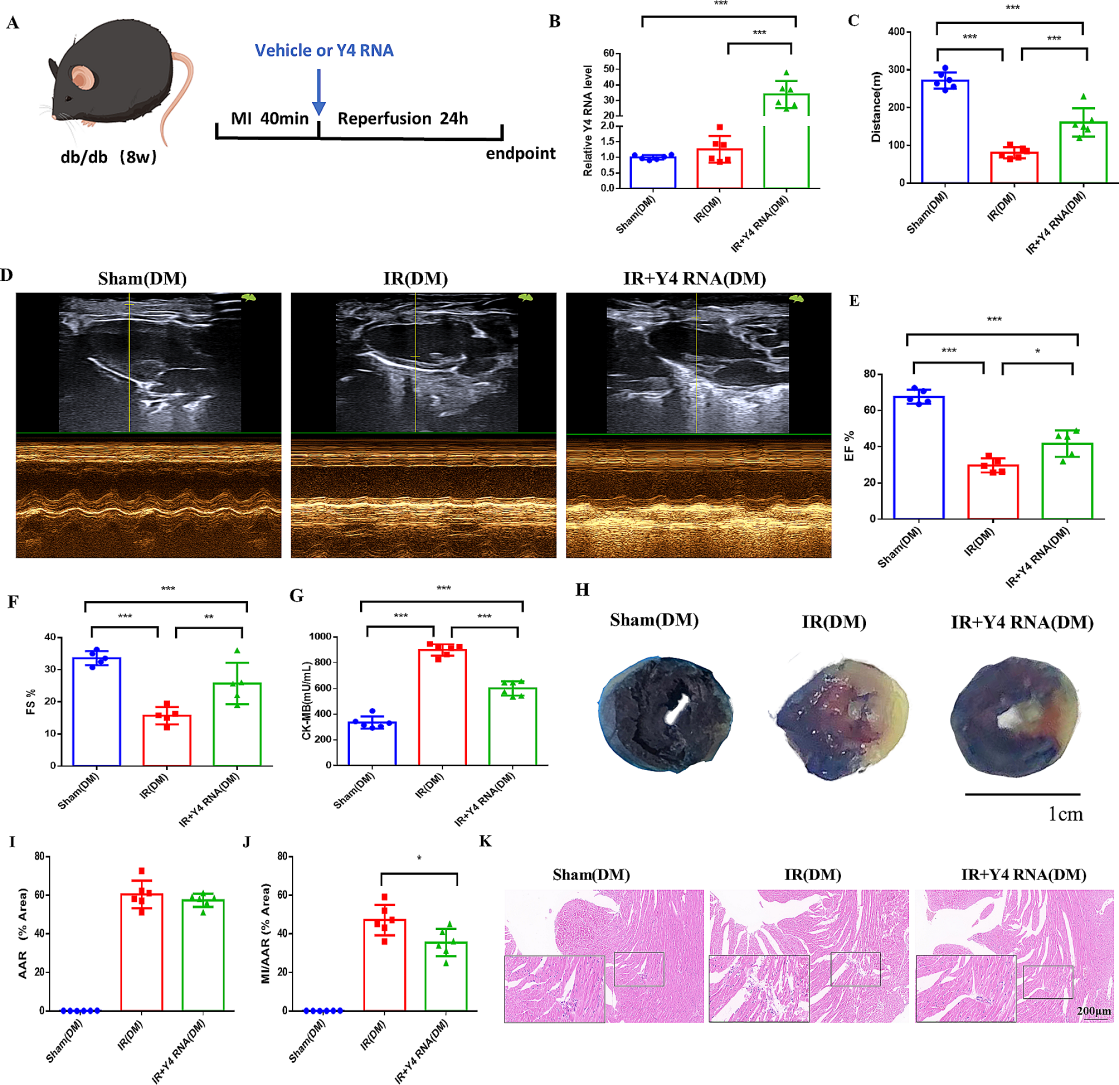
Y4 RNA improves cardiac function after myocardial I/R injury in db/db mice.(**A**) Flowchart of the Y4 RNA intervention experiment in the DM mouse myocardial I/R injury model. (**B**) qPCR was used to detect the abundance of Y4 RNA after cardiac injection in each group of mice. (**C**) Distance walked on a treadmill in each group of mice. (**D**) Representative M-mode echocardiographic images of cardiac function in each group of mice. (**E-F**) Left ventricular ejection fraction (EF) and left ventricular shortening (FS). (**G**) ELISA was used to detect the plasma levels of CK-MB in mice. (**H**) Evans blue/TTC-stained images showing the myocardial infarct area (white staining) in mice. (**I**) Hazardous area was defined as the unstained blue area as a percentage of the left ventricular area, and the difference between groups was not statistically significant. (**J**) The infarcted area was defined as the percentage of white area in the AAR. (**K**) Micrographs of morphological changes in the myocardium of mice in each group stained with H&E (magnification: ×10), with rectangles defining areas of higher magnification (×40). *P < 0.05, **P < 0.01, ***P < 0.001; *n* = 6 mice/group

To examine the impact of Y4 RNA derived from CDCs outer vesicles on diabetic myocardial I/R injury, a myocardial I/R injury model was established in db/db mice. Following 40 min of cardiac ischemia, the occlusion was released to reestablish coronary blood flow. Subsequently, 100 µl of Y4 RNA was administered into the left ventricular cavity for 20 s under an aortic cross-clamp, with a comparable dose of Dharmafect transfection reagent being administered to the sham and control groups (Fig. 2A). To assess the effectiveness of myocardial injection, the expression of Y4 RNA was examined 24 h post injection. The findings demonstrated a significant increase in Y4 RNA levels following myocardial injection (Fig. 2B). The findings of the treadmill experiment conducted 24 h postreperfusion indicated that Y4 RNA treatment led to a significant increase in maximal walking capacity on the treadmill compared to that in the I/R group (Fig. 2C). Echocardiographic assessments revealed a deterioration in cardiac function in the I/R group, while Y4 RNA treatment mitigated the decrease in the left ventricular shortening rate and ejection fraction following I/R injury (Fig. 2D-F). Furthermore, we conducted a comparison of cardiac function between the Sham group and the Sham group following Y4 RNA intervention. Our findings indicate that Y4 RNA intervention did not have a significant impact on cardiac function in db/db mice under non-ischemia/reperfusion conditions. (Supplemental Fig. 1A-C). To elucidate the extent of myocardial damage, CK-MB levels were measured in the blood of mice within each experimental group. The findings indicated that intervention with Y4 RNA mitigated myocardial damage (Fig. 2G). Additionally, to visually evaluate the severity of myocardial I/R injury, Evans blue/TTC staining was conducted on tissue samples from the ischemic region. The results revealed a significantly reduced area of myocardial infarction in the Y4 RNA group compared to the I/R group (Fig. 2H-J). Histological examination via HE staining revealed that the cardiomyocytes in the sham group exhibited uniform staining, preserved cellular architecture, and well-organized myocardial fibers. Conversely, cardiomyocytes in the I/R group displayed distinct infarct characteristics, including consolidation, disarrayed myocardial fibers, uneven staining, and increased cell lacunae. The myocardial damage observed in the Y4 RNA group was mitigated (Fig. 2K). Subsequently, TUNEL staining was conducted on tissue sections obtained from each cohort of mice to assess myocardial apoptosis. The findings revealed a marked elevation in the percentage of apoptosis-positive cells following myocardial I/R injury, whereas Y4 RNA intervention was observed to mitigate this increase (Supplemental Fig. 2A-B).

### Y4 RNA may attenuate the diabetic myocardial I/R injury inflammatory response through PKCβ/ERK1/2-mediated macrophage polarization

**Fig. 3:**
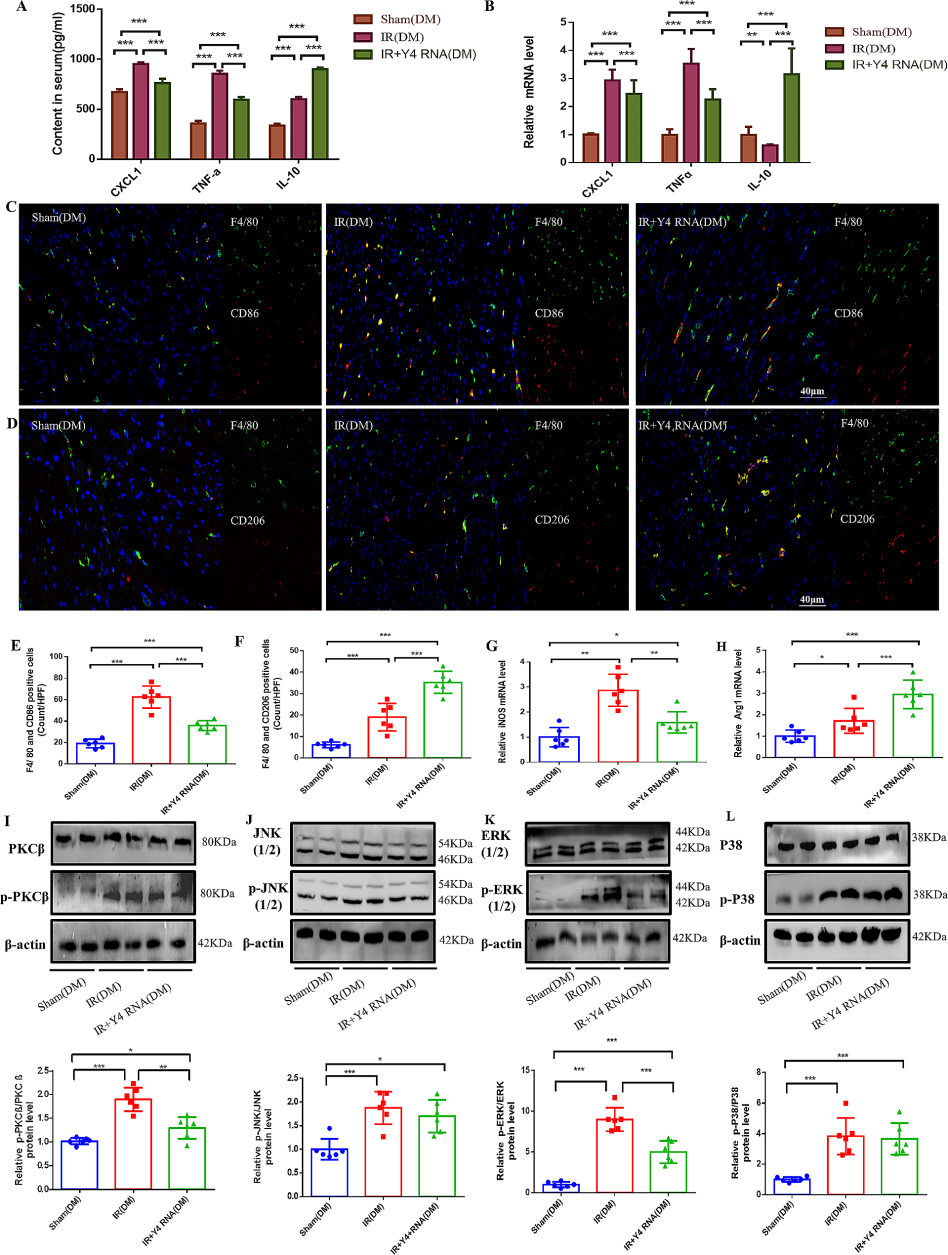
Y4 RNA may attenuate the diabetic MIRI inflammatory response through PKCβ/ERK1/2-mediated macrophage polarization. (**A**) ELISA was performed to measure the levels of CXCL1, TNF-α and IL-10 in the serum of DM mice in each group. (**B**) qPCR was performed to detect the expression of the CXCL1, TNF-α and IL-10 genes in the myocardial tissues of DM mice in each group. (**C-F**) Detection and quantification of M1 macrophages (F4/80+/CD86+) and M2 macrophages (F4/80+/CD206+) in mouse hearts by immunofluorescence. (**G-H**) qPCR was performed to determine iNOS (G) and Arg1 (H) gene expression in mouse myocardial tissues. (**I-L**) The levels of phosphorylated PKCβ, phosphorylated JNK1/2, phosphorylated ERK1/2, and phosphorylated P38 were detected by protein blotting. Quantification of protein blot band intensity is shown below the bands. *P < 0.05, **P < 0.01, ***P < 0.001; *n* = 6 mice/group

Y4 RNA has demonstrated ineffectiveness against isolated cardiomyocytes [16], but its immunomodulatory impact on macrophages has garnered attention from researchers. In this study, we investigated the involvement of Y4 RNA in cardiac inflammation following diabetic myocardial I/R. Analysis of serum samples from all mouse groups after 24 h of reperfusion revealed a significant reduction in CXCL1 and TNF-α levels in the Y4 RNA-treated group. Notably, there was a marked increase in the level of IL-10, a marker associated with M2 macrophages (Fig. 3A). qPCR analysis of mouse myocardial tissues revealed that the expression of CXCL1 and TNF-α increased following the induction of I/R injury, whereas Y4 RNA treatment led to a reduction in inflammatory factor expression and an increase in IL-10 expression (Fig. 3B). Immunofluorescent labeling was employed to identify M1 and M2 macrophages within the myocardium at the injury site, demonstrating a notable increase in the proportion of M2 macrophages after Y4 RNA intervention, despite no significant alteration in the total macrophage count (Fig. 3C-F). The PCR results similarly supported this conclusion (Fig. 3G-H). In order to investigate the potential interaction between Y4 RNA and PKCβ, we assessed the expression levels of PKCβ in myocardial tissues of mice across experimental groups. Our findings indicate that Y4 RNA suppresses the phosphorylation of PKCβ (Fig. 3I). In addition, using protein blotting to detect the expression of MAPK pathway-associated proteins, we found that Y4 RNA intervention inhibited the hyperphosphorylation of ERK1/2 but had no significant effect on JNK1/2 or P38 (Fig. 3J-L).

### Y4 RNA can ameliorate cardiomyocyte injury under high-glucose hypoxia-reoxygenation conditions

**Fig. 4:**
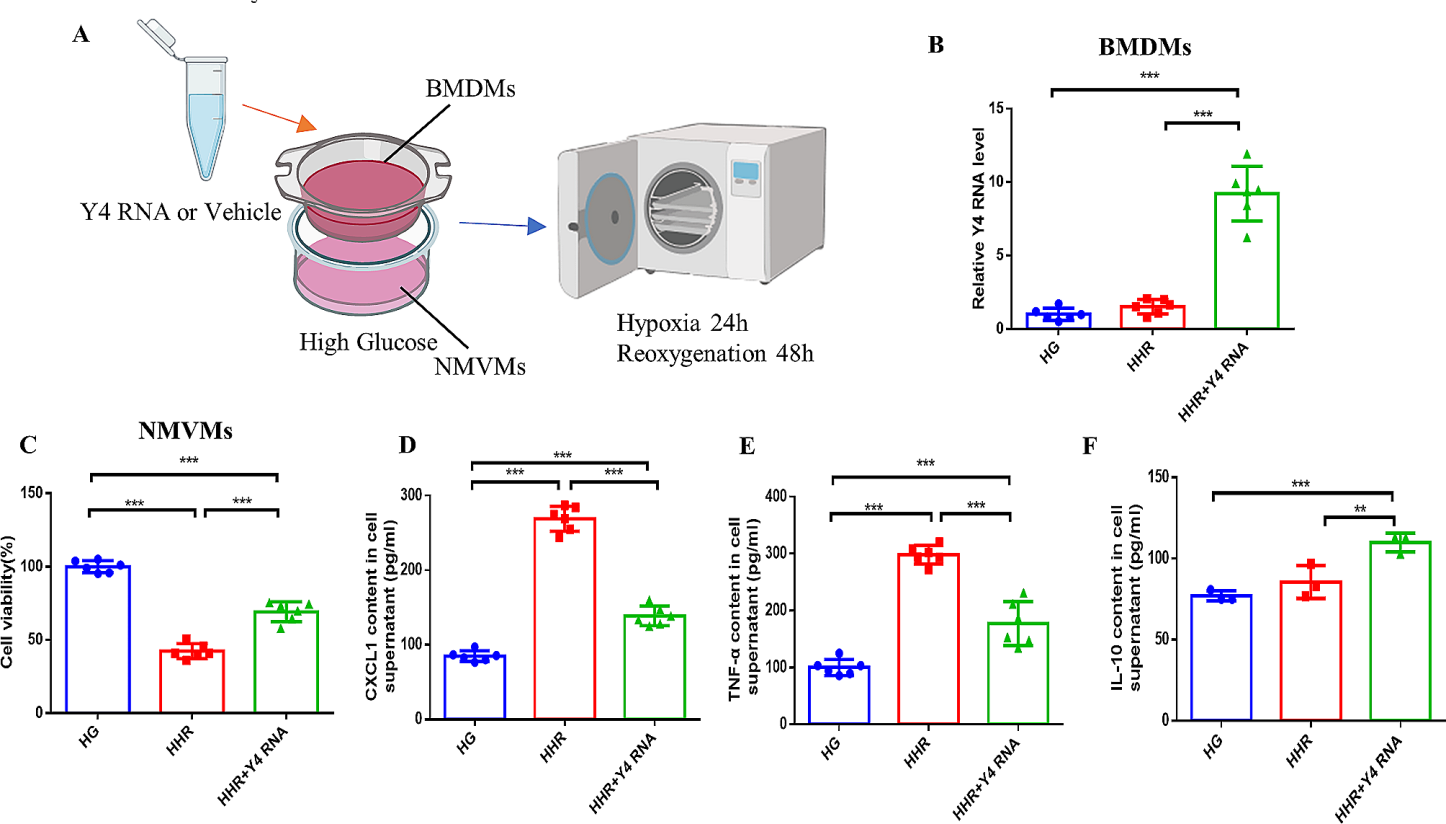
Y4 RNA can ameliorate cardiomyocyte injury under high-glucose hypoxia-reoxygenation conditions. (**A**) According to the experimental flow chart, Y4 RNA-treated BMDMs in a high-glucose environment were cocultured with NMVMs and subjected to hypoxia reoxygenation.(**B**) The rate of Y4 RNA transfection in macrophages was detected by qPCR. (**C**)The viability of NMVMs was detected by CCK-8. (**D-F**) ELISA was performed to measure the levels of CXCL1 (C), TNF-α (D), and IL-10 (E) in the cell cultures of each cell group. *P < 0.05, **P < 0.01, ***P < 0.001; *n* = 6 replicates/group

The evidence that Y4 RNA regulates macrophage activity is well established. Here, we cultured NMVMs and BMDMs in high-sugar medium and transfected the macrophages with vehicle or Y4 RNA. The cocultured cells were then placed in a hypoxic chamber to simulate a high-sugar hypoxia-reoxygenation (HHR) environment (Fig. 4A). In order to evaluate the impact of Y4 RNA transfection on macrophages, the expression of Y4 RNA within the macrophages was analyzed 24 h post-transfection. The findings indicated a significant elevation in the levels of Y4 RNA within the macrophages following transfection (Fig. 4B). The experimental results showed that cardiomyocyte viability was significantly lower after HHR than after nonhypoxic reoxygenation, whereas the viability of NMVMs cultured with Y4 RNA improved (Fig. 4C). Moreover, the expression levels of CXCL1 and TNF-α were significantly decreased, whereas the content of the anti-inflammatory factor IL-10 was increased (Fig. 4D-F).

### Y4 RNA may act directly on the PKCβ/ERK 1/2 signaling pathway in macrophages to induce M2 macrophage polarization

**Fig. 5:**
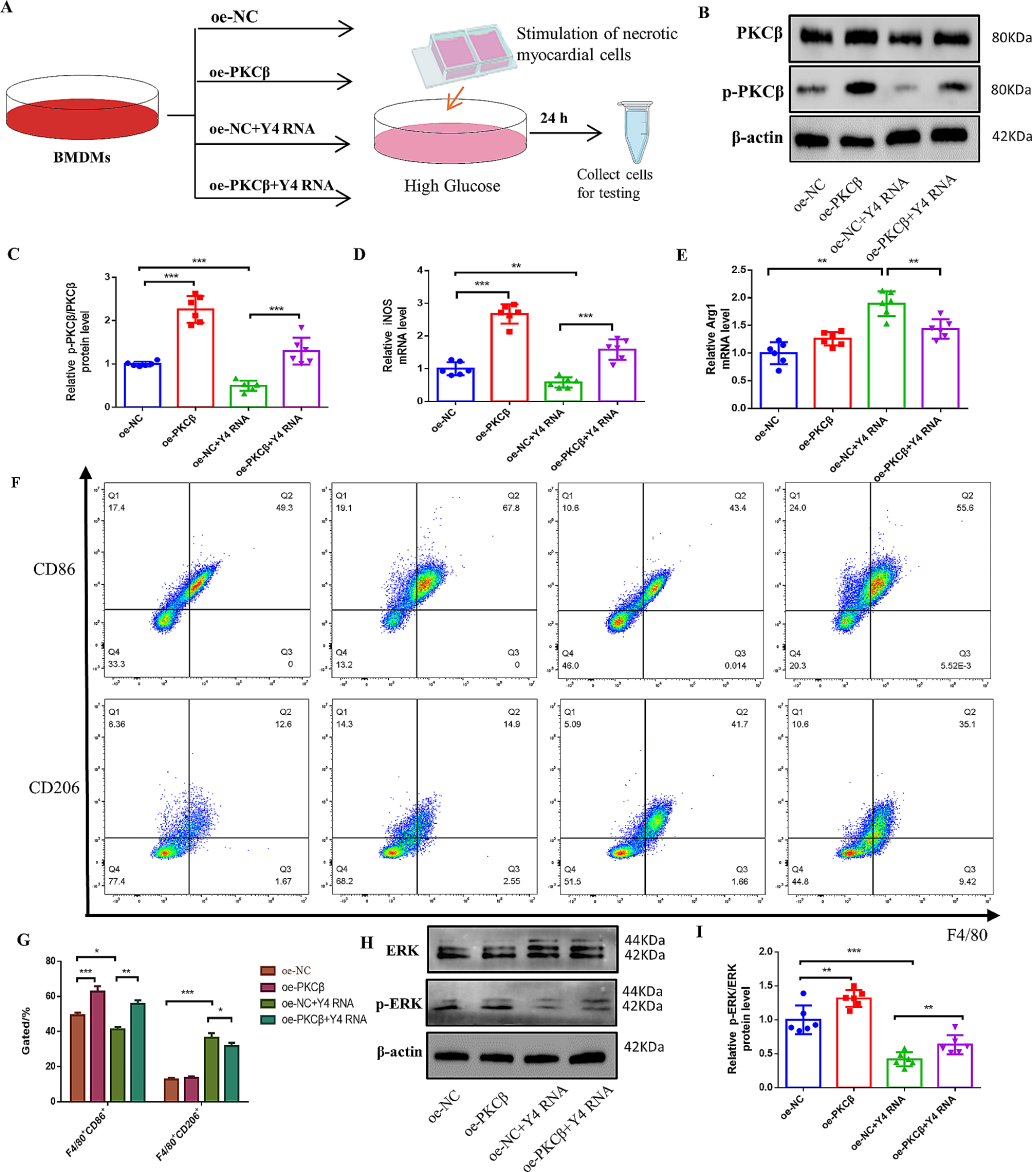
Y4 RNA may act directly on the PKCβ/ERK 1/2 signaling pathway in macrophages to induce M2 macrophage polarization.(**A**) Necrotic cardiomyocyte stimulation treatments for each group of BMDMs transfected with Y4 RNA according to the experimental workflow diagram. (**B**) Representative protein blots for PKCβ. (**C**)Quantitative data showing the PKCβ protein concentration in each group of BMDMs. (**D-E**) qPCR analysis of the gene expression of iNOS (D) and Arg1 (E) in BMDMs. (**F**) Representative pseudocolor flow cytometry showing the distribution of CD86 + and CD206 + macrophages. (**G**) Proportion of CD86 + and CD206 + macrophages in each group of BMDMs. H. Representative bands of phosphorylated ERK1/2 determined by protein blotting. (**I**) Quantitative data showing the protein expression of ERK1/2 in BMDMs. *P < 0.05, **P < 0.01, ***P < 0.001; *n* = 6 replicates/group

Building upon the findings from animal studies, our investigation sought to confirm the protective effects of Y4 RNA on cardiomyocytes through its influence on PKCβ expression in macrophages and subsequent phosphorylation of the ERK signaling pathway. Following the transfection of Y4 RNA into BMDMs overexpressing PKCβ in a high-glucose environment, necrotic cardiomyocyte supernatant was added to the culture medium to induce the activation of BMDMs (Fig. 5A). Compared with that in the oe-NC group, PKCβ expression in BMDMs following transfection with oe-PKCβ was notably upregulated. Treatment of oe-NC cells with Y4 RNA led to a significant decrease in PKCβ expression; however, this intervention was insufficient to fully counteract the effects of PKCβ overexpression. Nevertheless, Y4 RNA was still able to inhibit PKCβ activity (Fig. 5B-C). Subsequently, we conducted additional experiments to confirm the impact of Y4 RNA on macrophage polarization following oe-PKCβ treatment. The overexpression of PKCβ in BMDMs stimulated by necrotic cardiomyocytes notably skewed them toward the M1 phenotype, while Y4 RNA was observed to enhance the polarization of BMDMs toward the M2 phenotype. However, this effect was diminished upon PKCβ overexpression (Fig. 5D-G). Additionally, our findings indicated that Y4 RNA mitigated ERK1/2 hyperphosphorylation in BMDMs, although it was insufficient to counteract the influence of PKCβ overexpression (Fig. 5H-I).

### PKCβ deficiency attenuates inflammatory responses to myocardial I/R and inhibits cardiac macrophage polarization to the M1 phenotype in mice

**Fig. 6:**
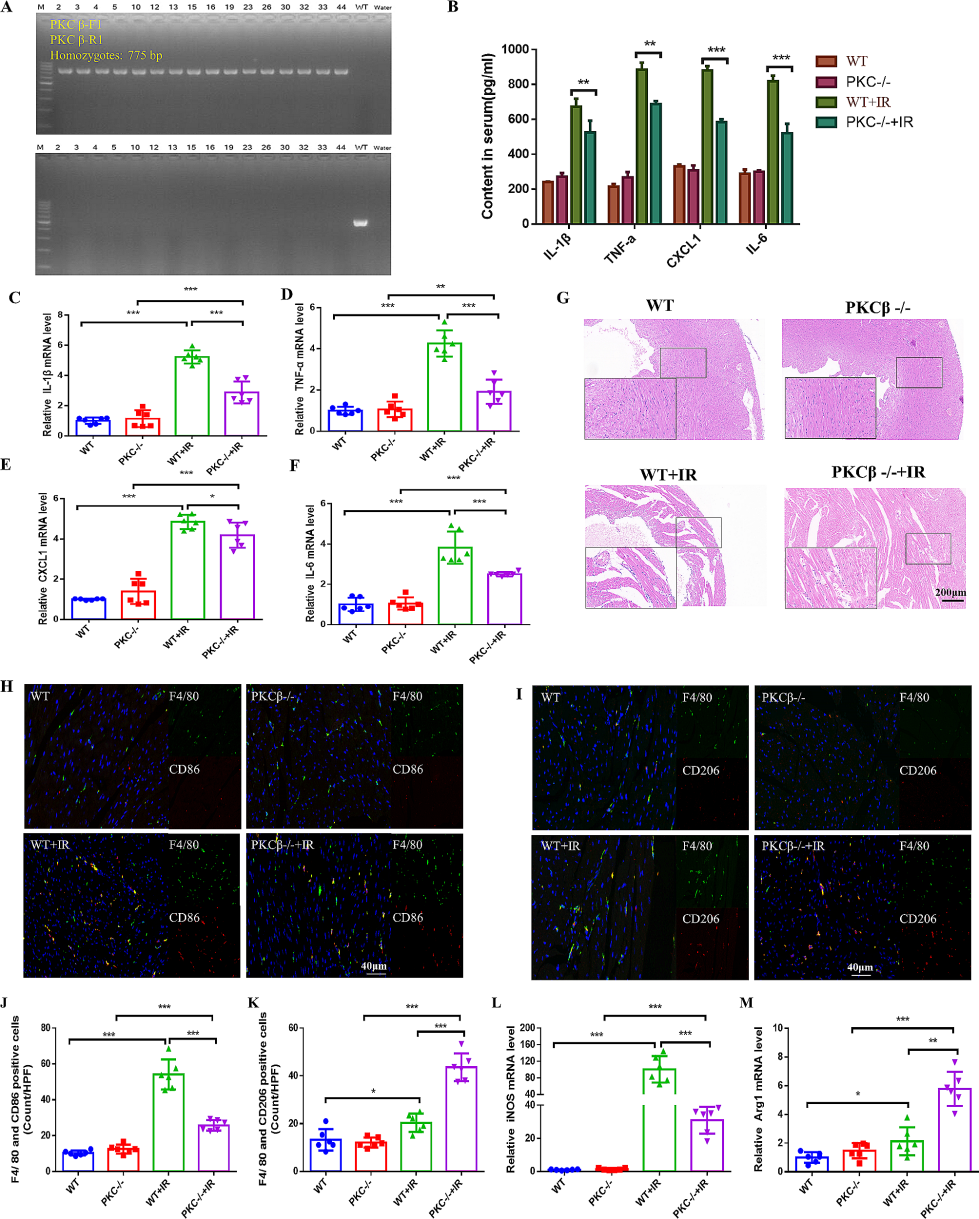
PKCβ deficiency attenuates inflammatory responses to myocardial I/R and inhibits cardiac macrophage polarization to the M1 phenotype in mice.(**A**) Characterization of PKCβ knockout mice.(**B**) ELISA was performed to measure the levels of CXCL1, TNF-α and IL-10 in the serum of mice in each group. (**C-F**) Quantitative PCR analysis of the gene expression levels of IL-1β (**C**), TNF-α (**D**), CXCL1 (**E**), and IL-6 (**F**) in myocardial tissues from WT and PKCβ -/- mice at 24 h after sham operation or I/R injury.(**G**) Micrographs of morphological changes in the myocardium of mice in each group stained with H&E (magnification: ×10), with rectangular boxes defining areas of higher magnification (×40). (**H-K**) Immunofluorescence detection and quantification of M1 macrophages (F4/80+/CD86+) and M2 macrophages (F4/80+/CD206+) in the hearts of mice in each group 24 h after I/R. (**L-M**) Quantification of the gene expression of iNOS (L) and Arg1 (M) in the myocardial tissues of WT and PKCβ-/- mice after the sham operation or I/R, as determined by qPCR. *P < 0.05, **P < 0.01, ***P < 0.001; *n* = 6 mice/group

Prior studies have shown that Y4 RNA suppresses PKCβ activation, leading to a reduction in the inflammatory response caused by ischemia/reperfusion injury in diabetic individuals. Nevertheless, the impact of PKCβ activation on macrophage polarization remains uncertain. In order to provide further substantiation for the aforementioned assertion, we utilized PKCβ knockout (PKCβ-/-) mice (Fig. 6A) to induce myocardial ischemia/reperfusion injury and compared the outcomes with WT mice. The findings from the tests conducted on the sera of different groups of mice indicated that the absence of the PKCβ gene had no impact on the levels of inflammatory factors in the serum of healthy mice (Fig. 6B). Nevertheless, following the induction of I/R, the absence of PKCβ resulted in a reduction of inflammatory factors in the serum. This observation was further supported by the qPCR results obtained from myocardial tissue (Fig. 6C-F). The findings from echocardiography and histological examination indicate that the absence of PKCβ does not have a significant impact on cardiac function and structure in mice under basal conditions. Conversely, downregulation of the PKCβ gene has been shown to enhance cardiac function following ischemia/reperfusion injury, thereby mitigating myocardial damage (Supplemental Fig. 3A-C and Fig. 6G).Furthermore, the deletion of the PKCβ gene resulted in the polarization of macrophages toward the cardiac M2 phenotype, characterized by decreased CD86 expression and increased CD206 expression (Fig. 6H-K). Specifically, the expression of M1-specific iNOS decreased, while the expression of M2-specific Arg1 increased in myocardial tissue (Fig. 6L-M). These findings suggest that PKCβ deficiency may mitigate myocardial I/R injury and dampen the cardiac inflammatory response in mice. It is possible that PKCβ plays a role in mediating macrophage polarization, with polarization toward a proinflammatory phenotype exacerbating I/R injury.

## Discussion

Chronic tissue inflammation has been identified as a significant contributor to the unfavorable prognosis of myocardial I/R injury in individuals with diabetes [20]. The recruitment, aggregation, and activation of proinflammatory macrophages in metabolic tissues are proposed to be the primary mechanisms driving this persistent low-grade inflammation [21]. Notably, chronic activation of PKCβ is highlighted as a key inflammatory process in this context [22]. Research involving pharmacological inhibitors and peptide inhibitors has demonstrated that small molecule inhibitors targeting PKCβ effectively prevent cholesterol accumulation in activated macrophages.

This finding suggested that PKCβ plays a key role in foam cell formation [23]. A separate investigation demonstrated that LY333531, by targeting PKCβ, effectively suppressed the immune maturation of dendritic cells induced by diabetic atherosclerosis, mitigated chronic low-grade inflammation in diabetes, and promoted the stabilization and reduction of atherosclerotic plaques. Nevertheless, the potential impact of hyperglycemia and sustained activation of PKCβ on macrophages in diabetic individuals, as well as their potential exacerbation of myocardial I/R injury, remains unexplored [24].

**Fig. 7:**
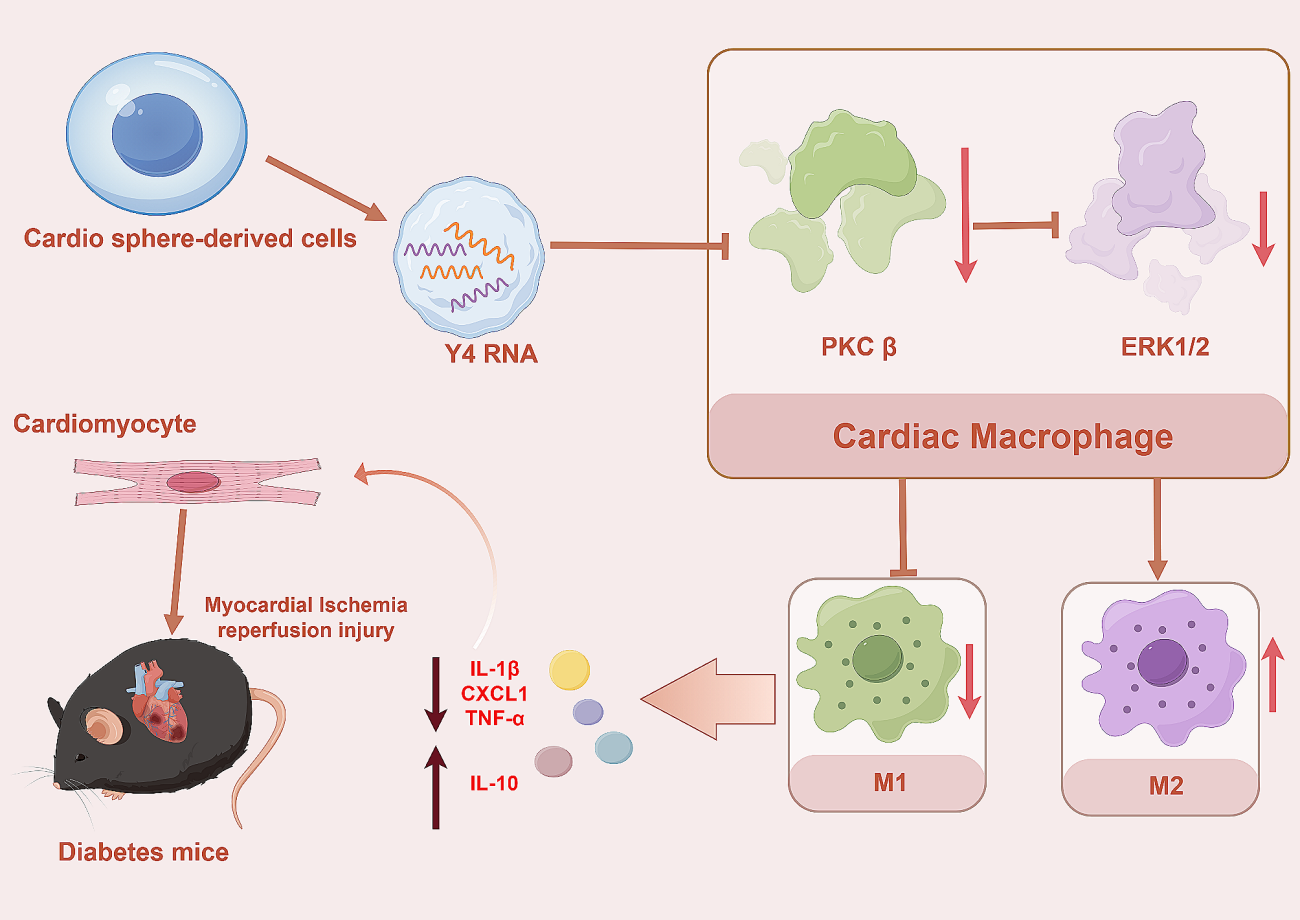
Y4 RNA is the most abundant small RNA species in CDC extracellular vesicles. Following diabetic myocardial I/R-induced cardiac inflammation, Y4 RNA promotes macrophage-induced differentiation to an anti-inflammatory phenotype and reestablishes an anti-inflammatory state through the inhibition of PKCβ/ERK1/2, thereby attenuating myocardial injury and improving cardiac function

This study demonstrated that the deletion of PKCβ following the initiation of myocardial I/R in nondiabetic mice mitigated myocardial damage and reduced cardiac inflammation by suppressing the proinflammatory polarization of macrophages. Additionally, our validation experiments revealed that PKCβ expression is upregulated following myocardial I/R in db/db mice, leading to worsened inflammation and an increase in M1 macrophages. In our investigation of novel therapeutic strategies, we discovered that Y4 RNA, the predominant RNA variant present in the extracellular vesicles of CDCs, can modulate the PKCβ/ERK1/2 signaling cascade in macrophages, thereby exerting a favorable effect on diabetic myocardial I/R injury (Fig. 7).

Over the past decade, CDCs have emerged as a promising therapeutic intervention for conditions such as myocardial infarction, dilated cardiomyopathy, dystrophic cardiomyopathy, and hypoplastic left heart syndrome. Early clinical studies have demonstrated the safety of CDCs in these contexts. Geoffrey de Couto et al. reported that CDCs may confer acute cardioprotection by inducing macrophage recruitment [25]. Cambier et al. identified Y4 RNA fragments within CDC exosomes that exhibit anti-inflammatory properties [16]. Their research demonstrated that Y4 RNA effectively decreased the presence of infiltrating CD68 + macrophages in myocardial tissues and modulated the secretion of the anti-inflammatory cytokine IL-10 by macrophages, thereby safeguarding myocardial cells against oxidative stress injury induced by H_2_O_2_ [26].

In this study, we present evidence of the beneficial impact of Y4 RNA administration on diabetic myocardial I/R injury. Our findings indicate a significant improvement in cardiac function, as well as a reduction in myocardial infarct size and inflammatory cell infiltration following intraventricular chamber delivery of Y4 RNA in db/db mice subjected to myocardial I/R injury. Extending previous studies, we observed that Y4 RNA decreased the expression of the proinflammatory cytokines CXCL1 and TNF-α and increased the expression of the anti-inflammatory factor IL-10 in diabetic I/R hearts. Our findings suggest that the increase in IL-10 may be a direct or indirect effect of Y4 RNA on macrophage polarization in the injured heart, inhibiting M1 macrophage production while promoting M2 macrophage production.

Recent research has demonstrated that the administration of CDCs to individuals with refractory heart failure results in the specific reversal of PKCβ upregulation. CDCs promote the phosphorylation of Myom1, Mybpc3, Des, and Vcl, and several of these phosphorylation sites are recognized as targets of PKC [6]. The observed phosphorylation events may be a component of a signaling cascade initiated by CDCs that ultimately leads to the downregulation of PKC. Our findings suggest that Y4 RNA may play a significant role in the inhibition of PKCβ upregulation induced by CDCs. We found that Y4 RNA treatment of db/db mice subjected to myocardial I/R similarly suppressed PKCβ expression.

In a streptozotocin-induced diabetes model, the activation of ERK1/2 myocardial phosphorylation during myocardial ischemia frequently occurs concurrently [27]. Likewise, cardiac dysfunction induced by obesity/insulin resistance is linked to heightened S6 kinase 1 and ERK1/2 signaling, while elevated JNK signaling in the diabetic myocardium exacerbates oxidative stress, endoplasmic reticulum stress, and interstitial fibrosis [28, 29]. Evidence suggests that PKC isoforms can activate the MAPK pathway through various mechanisms, such as Ras/Raf, which mediates the upregulation of inflammatory cytokines by macrophages during atherogenesis through the ERK signaling pathway and promotes ox LDL uptake and foam cell formation [30, 31]. Interestingly, our evidence showed that Y4 RNA had nonsignificant effects on JNK and P38 in the MAPK signaling pathway in diabetic myocardial I/R and merely attenuated ERK1/2 phosphorylation.

In vitro experiments revealed that Y4 RNA does not exert a direct effect on damaged cardiomyocytes. However, it was found that Y4 RNA can indirectly influence cardiomyocytes cocultured with BMDMs by modulating macrophage secretion through a cytokine profile, ultimately enhancing cardiomyocyte viability. In pursuit of this objective, we conducted a detailed investigation into the mechanisms by which Y4 RNA regulates macrophages. Our findings indicate that Y4 RNA intervention in macrophages stimulated by necrotic cardiomyocytes results in the inhibition of PKCβ expression and ERK1/2 phosphorylation in activated macrophages, ultimately influencing macrophage polarization toward the M2 phenotype. In our study involving Y4 RNA intervention in macrophages transfected with adenovirus overexpressing PKCβ, we observed that elevated PKCβ expression may impede the regulatory influence of Y4 RNA on macrophages relative to those not overexpressing PKCβ. These findings underscore the direct involvement of Y4 RNA in macrophage polarization via the PKCβ/ERK1/2 signaling pathway and underscore the significance of intercellular communication in cardiac repair processes.

Despite the notable findings of this research, it is important to acknowledge certain limitations and deficiencies. Specifically, the experimental design involving animal subjects focused solely on a single time point, specifically 24 h post myocardial I/R injury in mice, to investigate the mechanism of action of Y4 RNA. Subsequent investigations will prioritize the examination of the temporal aspect’s impact on experimental outcomes and endeavor to incorporate a time gradient analysis. Furthermore, the Y4 RNA utilized in this research was artificially synthesized; thus, the consistency of its functionality within the extracellular vesicles of CDCs in vivo remains unconfirmed. It is imperative to improve the extraction and synthesis of Y4 RNA. Additionally, PKCβ exists in two distinct isoforms, βI and βII; however, the relationship between Y4 RNA and these isoforms was not investigated further in the present study. It is crucial to acknowledge that a significant portion of this research was carried out using mouse and cellular models, thus necessitating additional validation in human subjects to substantiate the significance and practicality of the results.

## Conclusion

In this study, we found that PKCβ is upregulated in diabetic myocardial I/R injury and exacerbates the inflammatory response by modulating macrophage polarization. Y4 RNA fragments in CDC exosome vesicles can attenuate diabetic myocardial I/R injury by inhibiting macrophage PKCβ expression and attenuating ERK1/2 phosphorylation, which induces macrophage polarization toward the M2 type. These findings suggest that Y4 RNA encapsulated in CDC exosomes could serve as a promising therapeutic intervention for the management of diabetic myocardial I/R injury.

### Electronic supplementary material

Below is the link to the electronic supplementary material.

Supplementary Material 1.

## Data Availability

No datasets were generated or analysed during the current study.
