# Supplementary material for: Y4 RNA fragments from cardiosphere-derived cells ameliorate diabetic myocardial ischemia‒reperfusion injury by inhibiting protein kinase C β-mediated macrophage polarization
Source: Cardiovasc Diabetol. 2024 Jun 12;23:202. doi: 10.1186/s12933-024-02247-6 (PMC11170846; doi:10.1186/s12933-024-02247-6)
Supplement: Supplementary file 1 — Supplementary Material 1. [file 12933_2024_2247_MOESM1_ESM.docx]

**1. Methods**

**TUNEL staining**

The cell nucleus was stained with hematoxylin, while the cytoplasm was stained with eosin. Following dewaxing and rehydration, the sections were detected using the TDT-mediated dUTP Nick end Labeling (TUNEL) cell apoptosis assay kit’s instructions, where apoptotic nucleus appeared green, and normal nucleus appeared blue.

**2. Results**


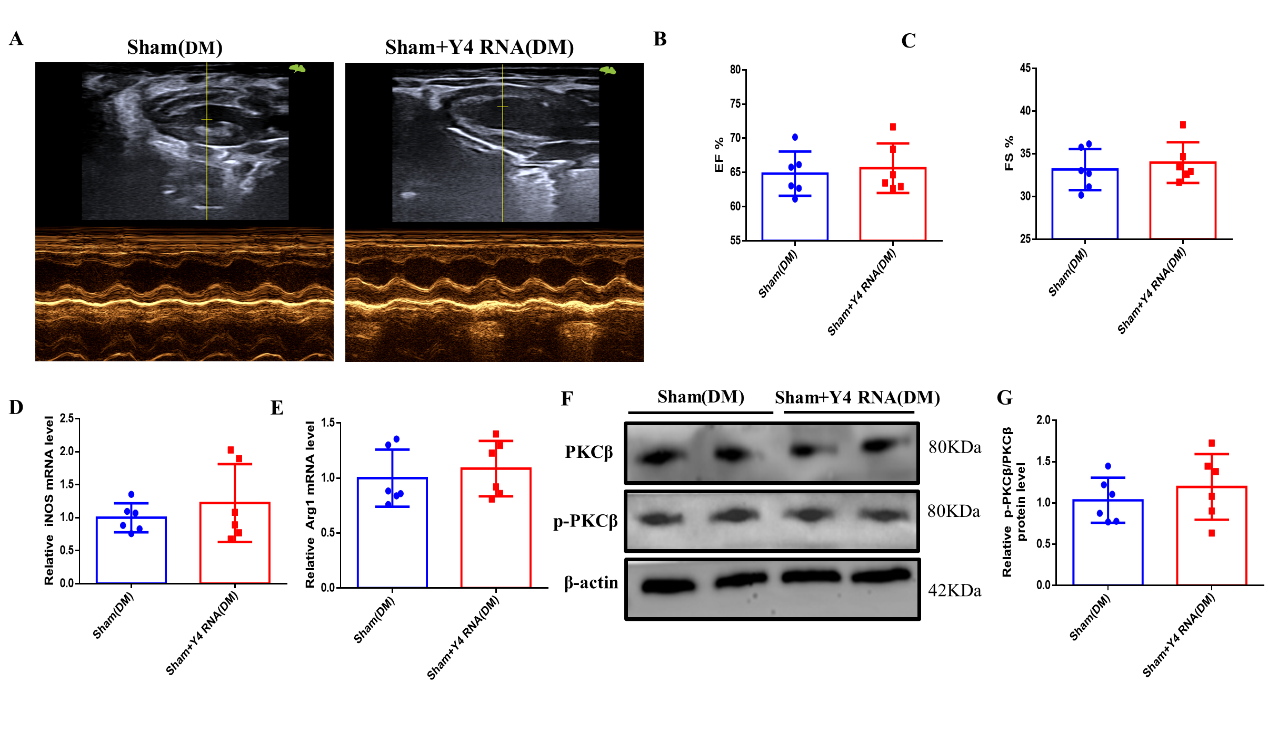


**Supplemental Figure 1**  Y4 RNA in the non-I/R state had no significant effect on cardiac function, macrophage polarisation and PKCβ expression in WT and db/db mice.

**(A)** Representative M-mode echocardiographic images of cardiac function in each group of mice. **(B-C)** Left ventricular ejection fraction (EF) and left ventricular shortening (FS). **(D-E)** qPCR was performed to determine iNOS (D) and Arg1 (E) gene expression in mouse myocardial tissues. **(F)** Representative protein blots for PKCβ.**(G)** Quantitative data showing the PKCβ protein concentration in each group. n = 6 mice/group


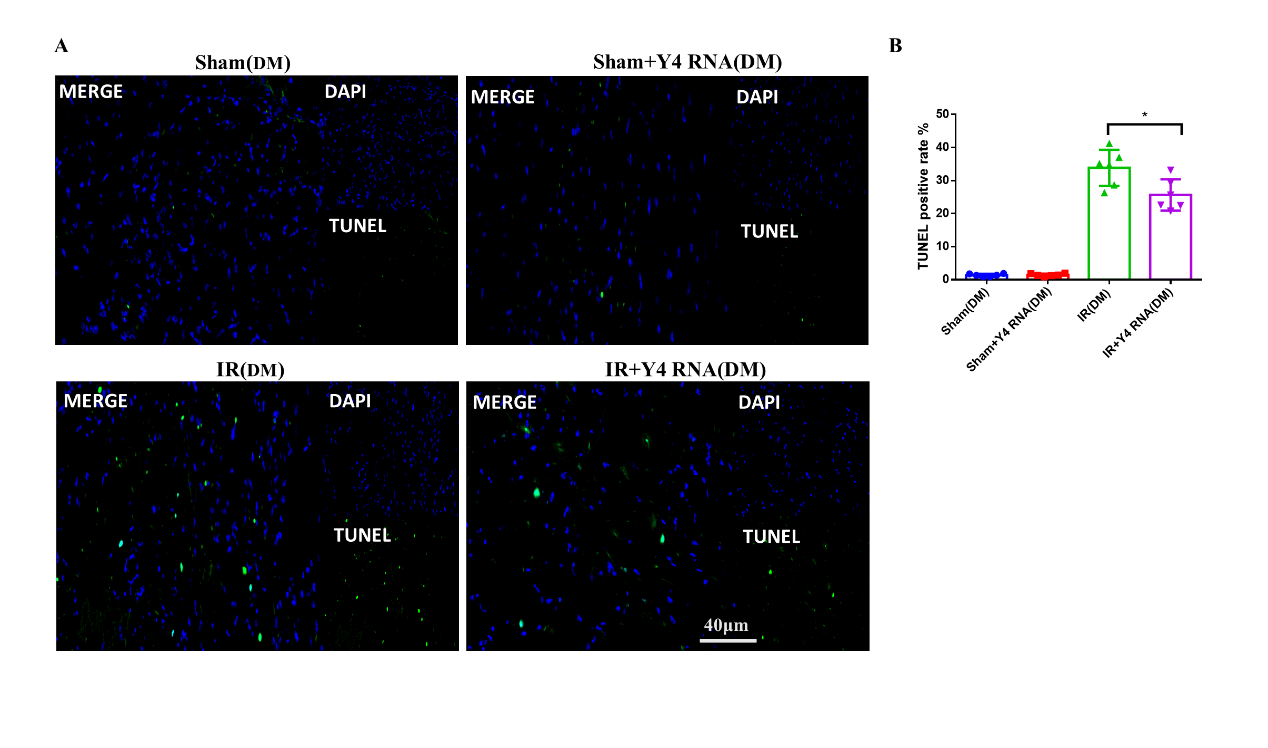


**Supplemental Figure 2** Y4 RNA reduces the proportion of apoptosis-positive cells.**(A)** Picture of TUNEL staining, the green dots indicate apoptotic cells, and the blue dots show nuclear. **(B)** The results of TUNEL fluorescence staining in each group. *P < 0.05

**
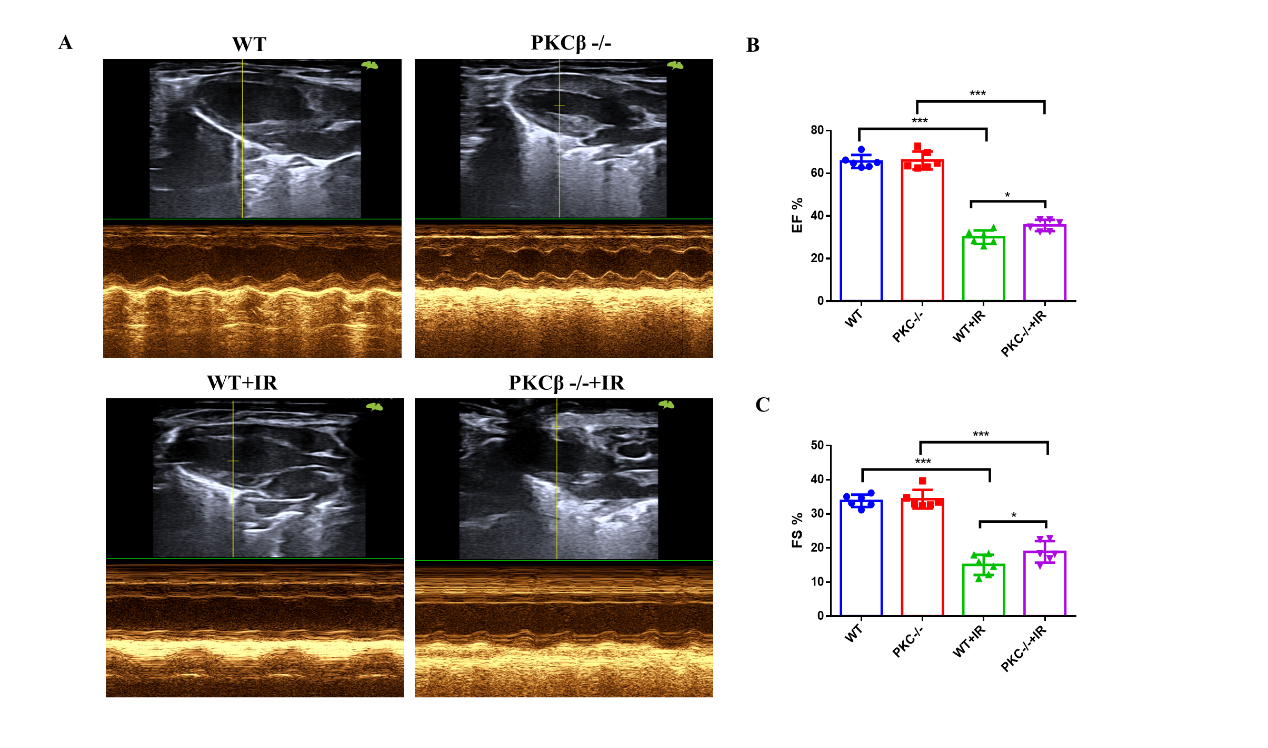
**

**Supplemental Figure 3** PKCβ knockdown did not affect cardiac function under nonischemic conditions.**(A)** Representative M-mode echocardiographic images of cardiac function in each group of mice. **(B-C)** Left ventricular ejection fraction (EF) and left ventricular shortening (FS). *P < 0.05,***P < 0.001; n = 6 mice/group
